# The effect of implantable collamer Lens V4c on ocular biometric measurements and intraocular lens power calculation based on Pentacam-AXL and IOLMaster 500

**DOI:** 10.1186/s12886-022-02644-z

**Published:** 2022-11-05

**Authors:** Di Zhang, Meng Yang, Ziyuan Liu, Hongyuan Cai, Xiaoyong Chen, Chun Zhang

**Affiliations:** 1 Department of Ophthalmology, Peking University Third Hospital, 49 North Garden Road, Haidian District, Beijing, 100191 PR China; 2 Beijing Key Laboratory of Restoration of Damaged Ocular Nerve, Peking University Third Hospital, 49 North Garden Road, Haidian District, Beijing, 100191 PR China; 3 Beijing Fenglian Jiayuelige Ophthalmic Clinic, 18 Chaoyangmenwai Street, Chaoyang District, Beijing, 100020 PR China

**Keywords:** ICL, Biometer, Intraocular lens power, Barrett universal II formula

## Abstract

**Background:**

To investigate the possible effect of implantable collamer lens (ICL) V4c on ocular biometric measurements by a new biometer Pentacam-AXL and partial coherence interferometry (PCI)-based IOLMaster 500 and intraocular lens power calculation using fourth-generation formula.

**Methods:**

We retrospectively enrolled patients who underwent ICL (EVO-V4c, STAAR Surgical Co. Nidau, Switzerland) implantation surgery from September 2020 to November 2021. The Pentacam-AXL and IOLMaster 500 biometers were used to measure axial length (AL), anterior chamber depth (ACD), keratometry (K), white to white (WTW), and central corneal thickness (CCT) values before and at least 2 months after ICL V4c implantation. The IOL power was calculated using the Barrett Universal II formula.

**Results:**

The study included 45 eyes in 28 patients. There was a significant increase in ALs (average 0.03 ± 0.07 mm, *p* = 0.01) and a significant decrease of ACDs (average 0.19 ± 0.17 mm, *p* < 0.001) based on Pentacam-AXL. Similar changes in ALs and ACDs were also found in IOLMaster 500. In addition, the difference in WTWs in the two devices and that of CCTs in Pentacam-AXL were statistically significant. However, the preoperative and postoperative K1 and K2 were separately comparable using either device. The IOL power calculated by the Barrett Universal II formula did not change significantly either by the software built in Pentacam-AXL or by manually putting the parameters of the IOLMaster 500 into the formula manually (*p* = 0.058, *p* = 0.675, respectively).

**Conclusions:**

Ocular parameters including ALs, ACDs, WTWs, and CCTs using a new Pentacam-AXL and standard PCI-based IOLMaster 500 changed significantly before and after the ICL V4c implantation, while IOL power prediction using the Barrett Universal II formula was little affected.

## Background

Implantable collamer lens (ICL), a posterior chamber phakic intraocular lens (PIOL), has been reported to perform well for correcting moderate to high myopia, with its good effectiveness, predictability and long-term safety [1–3]. With the growing popularity of ICLs in clinical use, studies investigating further possible issues have emerged in recent years. Although the postoperative complication of anterior subcapsular cataract with ICL V4c is vanishingly small [4, 5], the need for age-related cataract surgery with the large population of ICL-implanted patients is still unavoidable. Improvements in surgical techniques and IOL design and calculations have seen an increasing demand and expectations to achieve spectacle-free status. Thus, it is vital to understand whether the ICLs interfere the accuracy of ocular biometrics and consequently IOL power.

Previous studies have reported the influence of ICLs on ocular measurements and IOL calculations. Amro et al. evaluated the effect of ICL insertion on biometric parameters and IOL power calculations with third- and fourth-generation formulas based on IOLMaster 500 [6]. Chen et al. recently reported that even if the biometrics of anterior chamber depth (ACD) and lens thickness (LT) based on IOLMaster 700 and Sirius could be misrecognized, the IOL power has been little affected [7]. Other authors have studied the effect of ICL on axial length measurement with partial coherence interferometry (PCI) (IOLMaster 500, Carl Zeiss Meditec AG) [8–10].

Accurate and reliable measurements of ocular parameters and, consequently, IOL power calculations are essential. Currently, there are several biometers available on the market with different principles. Optical biometry is considered one of the most accurate methods of ocular biometry. The PCI represented by the IOLMaster 500 has been widely used for IOL power calculation [11]. In addition, the Scheimpflug imaging system represented by Pentacam and Sirius has also shown clinical advantages [12, 13]. The new Pentacam-AXL combines a rotating Scheimpflug camera, which provides a three-dimensional scan of the anterior segment of the eye, with PCI technology to obtain measurements of axial length (AL). With this new feature, this device can complete calculations of IOL power required in cataract and refractive surgery [12, 14]. However, in eyes implanted with ICLs, the difference between pre- and postoperative ocular biometrics using Pentacam-AXL has not been evaluated. With new formulas created to better predict IOL power, it remains unknown whether this new apparatus is affected by ICL implantation. Here, we compared ocular parameters and IOL power calculations using the Barrett Universal II formula before and after ICL implantation based on Pentacam-AXL and IOLMaster 500.

## Methods

We retrospectively collected 45 eyes in 28 patients who underwent ICL (EVO-V4c, STAAR Surgical Co. Nidau, Switzerland) implantation surgery from September 2020 to November 2021 in Beijing Fenglian Jiayuelige Ophthalmic Clinic. The inclusion criteria for the analysis were a diopter of spherical power (DS) between − 4.00 and − 20.00, a diopter of cylindrical power (DC) lower than − 5.00, anterior chamber depth (ACD) > 2.8 mm and endothelial cell count > 2000 cells/mm. The exclusion criteria were the presence of any eye diseases that may affect the accuracy of ocular biometrics, including corneal abnormalities such as ectasia, dystrophy or trauma, previous refractive surgery. This study adhered to the tenets of the Declaration of Helsinki.

### Preoperative and postoperative protocols

Before the surgery, each participant was evaluated by history-taking especially on ocular disease and trauma, comprehensive ocular examinations, including visual acuity, intraocular pressure with noncontact tonometry, routine fundus examination and slit-lamp biomicroscopy. Patients underwent ocular measurements of a new biometer Pentacam-AXL (Oculus, Germany) and a standard PCI-based biometer (IOLMaster 500, Carl Zeiss Meditec AG, Germany) preoperatively and at least 1 month after the surgery. The parameters obtained were AL, ACD, horizontal visible iris diameter (white to white, WTW), keratometry on the flattest corneal meridian (K1), and keratometry on the steepest corneal meridian (K2). Central corneal thickness (CCT) was also obtained by Pentacam-AXL. It should be noted that the ACD displayed here in Pentacam-AXL was the distance from the endothelium to the lens anterior surface. Remeasurement was performed if the image quality was not satisfactory.

Calculation of intraocular lens power, using the Barrett Universal II was performed. The results were obtained when the target refraction was set as 0. Since the software built in Pentacam-AXL only provides dioptric values with a constant interval of 0.5, the IOL power closest to emmetropization was chosen for each eye for consistency. The constants used were set for the Alcon SN60WF IOL (Alcon Laboratories, Inc).

### Surgical technique

All ICL implantation surgeries were performed by a specific experienced surgeon. Before the surgery, patients were given dilating and cycloplegic agents. Patients underwent surgery with local topical anesthesia using 0.4% oxybuprocaine hydrochloride eye drops (Benoxil, Santen; Osaka, Japan). The anterior chamber was filled with viscoelastic agent and the ICL introduced through a 3.2 mm clear corneal incision using the manufacturer’s injector cartridge (STAAR Surgical AG). After the loops of ICL were positioned into the posterior chamber, the viscoelastic was washed out of the anterior chamber using a balanced salt solution.

### Statistical analysis

Statistical analysis was performed by using SPSS (version 24.0; IBM SPSS Statistics, Armonk, NY, USA). Descriptive data are presented as mean ± standard deviation (SD) for continuous variables. A 2-tailed paired-sample *t* test was performed to compare the difference between preoperative and postoperative values. A *p* value less than 0.05 was considered statistically significant. Linear regression analysis was used to analyze the difference of IOL calculation.

## Results

There were 45 eyes of 28 patients (including 6 males, 22 females) who underwent ICL implantation surgery collected in our study, with a mean age of 28.36 ± 5.07 years (range from 21 to 40 years old). All patients regularly visited the hospital at 1 day, 1 week and 1 month postoperatively, and no intraoperative or early postoperative complications were observed. The average spherical equivalent of implanted ICLs was − 9.09 ± 3.16 D. There were 14 eyes implanted with TICLs, and 31 eyes implanted with ICLs. Table 1 shows the preoperative and postoperative visual acuity and refractions, as well as other ocular characteristics.

**Table 1:** The characteristics of 43 eyes underwent ICL implantation surgeries

	Preoperative	1-day postoperative	1-week postoperative	1-month postoperative
UDVA (LogMAR)	1.22 ± 0.27	0.008 ± 0.053	0.027 ± 0.209	−0.139 ± 0.366
BCVA (LogMAR)	0.001 ± 0.007	NA	NA	NA
DS (D)	−6.98 ± 5.02	0.62 ± 0.75	0.29 ± 0.64	0.35 ± 0.45
DC (D)	−0.99 ± 1.45	− 0.62 ± 0.54	− 0.48 ± 0.33	− 0.44 ± 0.32
SE (D)	−7.48 ± 4.79	0.31 ± 0.59	0.05 ± 0.60	0.13 ± 0.46
CED (cells/mm^2^)	2915.84 ± 286.29	2875.02 ± 261.87	2827.82 ± 278.17	2812.92 ± 259.06
IOP (mmHg)	16.38 ± 2.16	16.96 ± 2.90	15.98 ± 2.22	16.13 ± 2.26
Vault (μm)	NA	677.89 ± 252.01	651.97 ± 303.25	619.32 ± 244.26

*UDVA* uncorrected distance visual acuity, *BCVA* best corrected visual acuity, *DS* diopter of spherical power, *DC* diopter of cylindrical power, *SE* spherical equivalent, *CCT* central corneal thickness, *CED* corneal endothelial density, *IOP* intraocular pressure, *NA* not available

### Preoperative and postoperative parameters measured by Pentacam-AXL

The changes in biometric variables measured by Pentacam-AXL preoperatively and postoperatively are shown in Table 2. We found a significant increase of AL (26.67 ± 1.15 mm versus 26.70 ± 1.15 mm, *p* = 0.010) and a significant decrease in ACDs (3.19 ± 0.26 mm versus 3.00 ± 0.25 mm, *p* < 0.001). In addition, the WTWs and CCTs before and after the surgery showed significant differences (*p* = 0.010 and 0.002, respectively). However, the K flat and K steep measured by Pentacam-AXL did not change significantly after ICL implantation (*p* = 0.888 and 0.168, respectively).

**Table 2:** Comparison of preoperative and postoperative ocular biometrics of the anterior chamber based on Pentacam-AXL

	Preoperative	Postoperative	*p*
AL (mm)	26.67 ± 1.15	26.70 ± 1.15	0.010^*^
ACD (mm)	3.19 ± 0.26	3.00 ± 0.25	< 0.001^*^
K1 (D)	42.24 ± 1.25	42.24 ± 1.21	0.888
K2 (D)	43.47 ± 1.58	43.35 ± 1.49	0.168
WTW (mm)	11.68 ± 0.32	11.72 ± 0.32	0.010^*^
CCT (μm)	512.73 ± 36.21	515.24 ± 34.14	0.002^*^
IOL (D)	11.84 ± 2.65	11.73 ± 2.60	0.058

*AL* axial length, *ACD* anterior chamber depth, *K1* keratometry on the flattest corneal meridian, *K2* keratometry on the steepest corneal meridian, *WTW* white to white, *CCT* central corneal thickness, *IOL* intraocular lens, *D* diopter

^*^p < 0.05

The difference between preoperative and postoperative IOL power calculated by the Barrett Universal II embedded in the Pentacam-AXL system was not statistically significant (11.84 ± 2.65 D versus 11.73 ± 2.60 D, *p* = 0.058), although there was significant difference in ALs, ACDs, WTWs and CCTs.

**Fig. 1:**
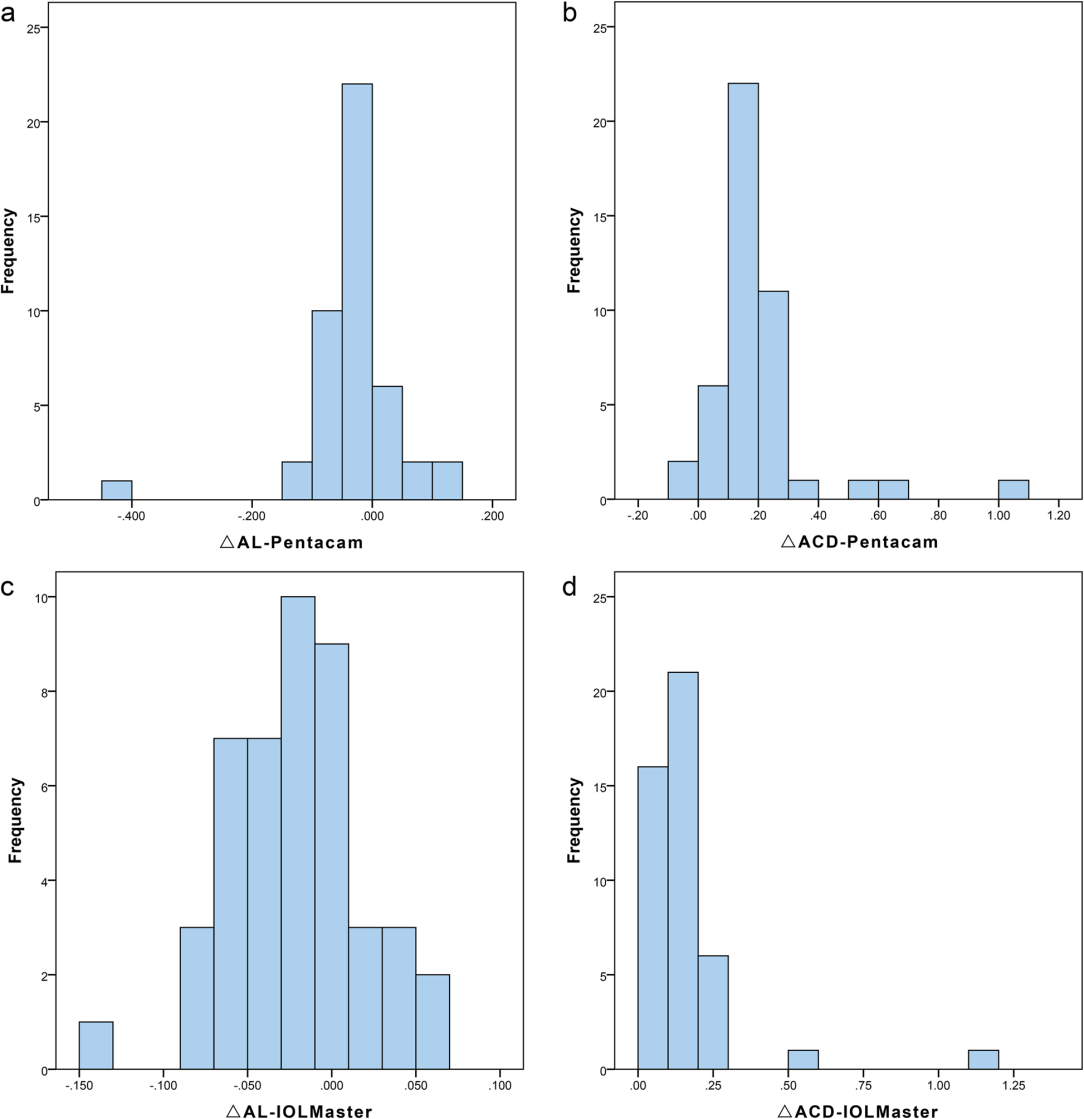
The distribution of the difference in AL and ACD pre- and postoperatively using Pentacam-AXL and IOLMaster 500. **a** The difference of AL in Pentacam-AXL; **b** the difference in ACD in Pentacam-AXL; **c** the difference in AL in IOLMaster 500; **d** the difference in ACD in IOLMaster 500; △ = pre-post

The distribution of the difference in AL and ACD between preoperatively and postoperatively is shown in Fig. 1(a, b). All results were calculated by subtracting postoperative data from preoperative data. The average difference of AL was − 0.03 ± 0.07 mm. It was obvious that only in one of 45 eyes was the AL difference significantly larger than that in the other eyes, which was 0.42 mm. There were 5 eyes (11.11%) with AL differences ≥0.1 mm and 40 eyes (88.89%) with AL differences less than 0.1 mm. Regarding the ACD difference, the average value was 0.19 ± 0.17 mm. Except for 2 eyes with a 0.01-mm increase in ACD, the other eyes all showed a decrease in ACD, which may not be explained simply by measuring errors.

### Preoperative and postoperative parameters measured by IOLMaster 500

A comparison of biometrics measured by IOLMaster 500 preoperatively and postoperatively is shown in Table 3. Similar to the results of Pentacam-AXL, there was a significant increase in AL (26.66 ± 1.14 mm versus 26.69 ± 1.15 mm, *p* < 0.001) and a significant decrease in ACD (3.73 ± 0.22 mm versus 3.57 ± 0.27 mm, *p* < 0.001) after the implantation of the ICL. In addition, the WTW showed significant difference (*p* = 0.010), while the K1 and K2 had no significant difference preoperatively and postoperatively (*p* = 0.203, 0.079, respectively), which is consistent with the results of Pentacam-AXL.

**Table 3:** Preoperative and postoperative ocular biometrics of the anterior chamber based on IOLMaster 500

	Preoperative	Postoperative	*p*
AL (mm)	26.66 ± 1.14	26.69 ± 1.15	< 0.001^*^
ACD (mm)	3.73 ± 0.22	3.57 ± 0.27	< 0.001^*^
K1 (D)	42.99 ± 1.31	42.94 ± 1.33	0.203
K2 (D)	44.44 ± 1.55	44.36 ± 1.54	0.079
WTW (mm)	12.18 ± 0.32	12.12 ± 0.33	0.010^*^
IOL (D)	11.50 ± 2.61	11.48 ± 2.64	0.675

*AL* axial length, *ACD* anterior chamber depth, *K1* keratometry on the flattest corneal meridian, *K2* keratometry on the steepest corneal meridian, *WTW* white to white, *IOL* intraocular lens, *D* diopter

^*^p < 0.05

After putting the ocular parameters of IOLMaster 500 into the Barrett Universal II formula, the difference between preoperative and postoperative IOL power was not statistically significant (11.50 ± 2.61 D versus 11.48 ± 2.64 D, *p* = 0.675).

The distribution of differences in AL and ACD based on IOLMaster 500 is displayed in Fig. 1(c, d). The average difference in ALs was − 0.03 ± 0.04 mm. There were 44 eyes with an AL difference < 0.1 mm, except for one eye > 0.1 mm. The average ACD difference was 0.15 ± 0.17 mm. As shown in the Fig. 1(d), all eyes underwent a decrease in the ACD.

### Linear regression of the IOL power difference using the Barrett universal II formula

The linear regression analysis showed the effect of each parameter on the difference of the IOL power (Table 4). In the results of Pentacam-AXL, the AL difference and K1 difference had significant effect on the difference in IOL power. However, incorporating all factors into the calculation, the IOL power based on Pentacam-AXL showed no significant change after ICL implantation. Regarding the IOLMaster 500, the difference in the K value showed a significant effect on the IOL power calculation.

**Table 4:** The Linear Regression of the IOL Power Difference Calculated by the Barrett Universal II Formula

	Factors	Unstandardized coefficients	Standard deviation	Standardized coefficients	*p*
IOL power based on Pentacam-AXL	AL difference	−1.921	0.623	− 0.390	0.004^*^
	ACD difference	0.112	0.280	0.052	0.692
	WTW difference	−0.262	0.503	−0.065	0.606
	K1 difference	−0.514	0.174	−0.418	0.006^*^
	K2 difference	−0.031	0.092	−0.046	0.739
	CCT difference	0.017	0.010	0.233	0.090
IOL power based on IOLMaster 500	AL difference	1.394	1.210	0.150	0.256
	ACD difference	−0.362	0.285	−0.171	0.212
	WTW difference	0.403	0.339	0.155	0.241
	K1 difference	−0.433	0.201	−0.304	0.037^*^
	K2 difference	−0.413	0.153	−0.343	0.010^*^

^*^p < 0.05

Considering that only one of the AL values measured by Pentacam-AXL was 0.42 mm, we reperformed the comparison of pre- and postoperative ALs by excluding this eye. However, the result was still significant (preoperative 26.66 ± 1.16 mm versus postoperative 26.69 ± 1.16 mm, *p* = 0.006). Interestingly, the linear regression analysis of 44 eyes showed that the AL difference had no significant effect on the IOL power (*p* = 0.167), while the other parameters measured by Pentacam-AXL showed similar results with 45 eyes (Table 5).

**Table 5:** The Linear Regression of the IOL Power Difference Measured by Pentacam-AXL in 44 Eyes

Factors	Unstandardized coefficients	Standard deviation	Standardized coefficients	*p*
AL difference	−1.422	1.007	−0.199	0.167
ACD difference	0.142	0.287	0.071	0.624
WTW difference	−0.222	0.512	−0.059	0.667
K1 difference	−0.513	0.176	−0.446	0.006^*^
K2 difference	−0.039	0.093	−0.063	0.678
CCT difference	0.017	0.010	0.244	0.102

^*^p < 0.05

## Discussion

The precise acquisition of ocular biometrics is the first step to correctly predict the IOL power calculation in refractive cataract surgery. Since a variety of advanced optical biometry instruments are available in clinical use, including PCI of IOLMaster 500, swept-source optical coherence tomography (SS-OCT) represented by IOLMaster 700 and Scheimpflug imaging system represented by Sirius and Pentacam-AXL, whether the existence of ICL in the optical pathway may alter the results of different measuring devices remains no consistent conclusion. The results of this study regarding the AL and ocular parameters of the anterior chamber by Pentacam-AXL and IOLMaster 500 showed deviations before and after ICL implantation. However, changes in AL, ACD, WTW and CCT did not make corrections to the IOL power calculation using the Barrett Universal II formula in the presence of ICL. To our knowledge, our study is the first to compare pre- and postoperative AL, anterior chamber parameters and IOL power calculations using Pentacam-AXL in patients implanted with ICLs.

Similar results have been reported in studies using other biometry. Previous studies compared the AL using PCI before and after ICL implantation and found that the small change in AL did not affect the IOL power calculation [6, 8, 9]. This could be explained by the high light transmittance and unique refractive gradient of the collamer, the main component of ICL, making its optical effect close to the crystalline lens. This implantable phakic lens has undergone several modifications, and the most widely used one, namely the V4c model came onto the market in 2011 [5]. This model incorporates a 0.36-mm central hole, thus making iridectomies or iridotomies unnecessary, maintaining normal aqueous flow, and still providing good and comparable optical quality with nonhole models [15, 16].

In this study, ALs were obtained by PCI whether using Pentacam-AXL or IOLMaster 500. As the same principle to measure AL was adopted in two biometers, there was significant difference between pre- and postoperative data using both devices. It has been reported that the IOLMaster 500 PCI biometer is less accurate for highly myopic eyes with a high AL [17]. Early vitreous liquefaction in eyes with axial myopia may change the refractive index of vitreous in long eyes, thus resulting in deviations of the optical path measurement of PCI [17]. However, patients undergoing ICL implantation are mostly high myopia with a long AL. This could be partly the derivation of the AL deviations in this study, while the effect of ICL on the accuracy of AL measurement could not be excluded completely. Several studies have shown that the IOLMaster 700 has better lens penetration ability and a higher success rate of AL measurement than PCI [11, 18]. IOLMater 700 is the first SS-OCT-based biometric device, enabling OCT imaging and visualization across the entire length of the eye [18]. Huang et al. suggested that SS-OCT-based biometers are likely to become the gold standard for AL measurement [19], but it remains unknown whether IOLMaster 700 has a better performance in the measurement of ALs in eyes with ICL. However, in this study, we performed the analysis of ALs without optimization and no significant change was found in the IOL calculations using the Barrett Universal II formula. Besides, the significant difference of ALs in both the devices did not significantly contributed to the IOL power difference. Given that the IOL in clinical use is usually designed as a 0.5-D interval, the results here only implied that the difference in IOL power had no clinical significance.

The mean decreases in ACD using Pentacam-AXL and IOLMaster 500 were 0.19 ± 0.17 mm and 0.15 ± 0.17 mm, respectively, in our study, which were both statistically significant (*p* < 0.001). Amro et al. found no change in ALs but a 0.27-mm decrease in ACDs was observed between pre- and post-operative values, which had no effect on the IOL power prediction in cases of high myopia using third- and fourth-generation formulas [6]. Chen et al. reported that the difference between preoperative and postoperative ACD was caused by the misidentification of the anterior surface of the crystalline lens [7]. This misidentification was observed in some eyes in the OCT images of IOLMaster 700 and was inferred to occur in eyes measured by Sirius. However, the IOL power calculated by Barrett universal II formula or ray-tracing technology showed no significant difference after ICL implantation [7]. Recently, Zhang et al. demonstrated the effect of ICL on the crystalline lens position [20]. Forward movement of the crystalline lens was found on day 1 postoperatively and was stable within 6 days [20]. This may help explain the difference in ACDs apart from the misidentification of the anterior surface of the lens. A decrease in ACD could change the IOL power calculation using the Hagis formula, which directly relies on the ACD value, while in other formulas the ACD value is averaged using other variables and constants [6]. In the Barrett Universal II formula, the relationship between the A-constant and the lens factor is also used to determine the ACD, which could help explain the nonsignificant difference in IOL power [21].

In the linear regression analysis, the insignificant K difference had contributed significantly on the IOL power difference in both the devices. We think that if the K value accounted for a large proportion in the formula, the IOL power would change significantly even if the K changed a little. In this study, a significant difference was found in WTW preoperatively and postoperatively with two instruments. We speculated that this deviation was due to the limbal scar of 3.2-mm clear corneal incision and measurement errors [22]. Since the value of WTW is optional in the Barrett Universal II formula^A^, the difference in WTW did not have influence on the IOL power calculation. However, Pentacam-AXL could not obtain the value of lens thickness in eyes with undilated pupils, which is also a parameter used in the newly developed formulas for IOL calculation such as the Barrett Universal II formula [23] and Kane formula [24].

This study has some limitations. First, the number of eyes enrolled in this study was relatively small; further study with more eyes would be desirable to corroborate the results. Second, only one formula with good predictability was used in this study; as advanced formulas based on artificial intelligence have recently been developed, more attention should be given to the effect of ICLs on the IOL power calculation using new formulas should be paid. Third, studies comprising instruments with different methods of measuring AL can be conducted to investigate their accuracy after ICL implantation.

## Conclusions

In conclusion, ocular parameters including ALs, ACDs, WTWs and CCTs using a new Pentacam-AXL and standard PCI-based IOLMaster 500 changed significantly before and after the ICL V4c implantation. However, the IOL power prediction using the Barrett Universal II formula was little affected. This could provide a reference that if the preoperative ocular biometrics could not be obtained, the postoperative data would be reliable to calculate IOL power using the Barrett Universal II formula based on both devices.

## Data Availability

The datasets generated and analyzed during the current study are available from the corresponding author on reasonable request following publication.

## Abbreviations

IOL: Intraocular lens
ICL: Implantable collamer lens
PCI: Partial coherence interferometry
UDVA: Uncorrected distance visual acuity
BCVA: Best corrected visual acuity
logMAR: Logarithm of the minimum angle of resolution
DS: Diopter of spherical power
DC: Diopter of cylindrical power
SE: Spherical equivalent
CED: Corneal endothelial density
IOP: Intraocular pressure
AL: Axial length
ACD: Anterior chamber depth
K1: Keratometry on the flattest corneal meridian
K2: Keratometry on the steepest corneal meridian
WTW: White to white
CCT: Central corneal thickness
D: Diopter
